# Vaccination With a Gamma Irradiation-Inactivated African Swine Fever Virus Is Safe But Does Not Protect Against a Challenge

**DOI:** 10.3389/fimmu.2022.832264

**Published:** 2022-04-26

**Authors:** Jutta Pikalo, Luca Porfiri, Valerij Akimkin, Hanna Roszyk, Katrin Pannhorst, Richard Thiga Kangethe, Viskam Wijewardana, Julia Sehl-Ewert, Martin Beer, Giovanni Cattoli, Sandra Blome

**Affiliations:** ^1^Institute of Diagnostic Virology, Friedrich-Loeffler-Institut, Greifswald - Insel Riems, Germany; ^2^Animal Production and Health Laboratory, Joint FAO/IAEA Centre of Nuclear Techniques in Food and Agriculture, International Atomic Energy Agency (IAEA), IAEA Laboratories, Seibersdorf, Austria; ^3^Chemical and Veterinary Investigations, Office Stuttgart, Fellbach, Germany; ^4^Institute of Molecular Virology and Cell Biology, Friedrich-Loeffler-Institut, Greifswald - Insel Riems, Germany; ^5^Department of Experimental Animal Facilities and Biorisk Management, Friedrich-Loeffler-Institut, Greifswald - Insel Riems, Germany

**Keywords:** African swine fever virus, gamma irradiation, inactivated vaccine, adjuvant, efficiency, domestic pigs

## Abstract

African swine fever (ASF) is among the most devastating viral diseases of pigs and wild boar worldwide. In recent years, the disease has spread alarmingly. Despite intensive research activities, a commercialized vaccine is still not available, and efficacious live attenuated vaccine candidates raise safety concerns. From a safety perspective, inactivated preparations would be most favourable. However, both historical and more recent trials with chemical inactivation did not show an appreciable protective effect. Under the assumption that the integrity of viral particles could enhance presentation of antigens, we used gamma irradiation for inactivation. To this means, gamma irradiated ASFV “Estonia 2014” was adjuvanted with either Polygen™ or Montanide™ ISA 201 VG, respectively. Subsequently, five weaner pigs per preparation were immunized twice with a three-week interval. Six weeks after the first immunization, all animals were challenged with the highly virulent ASFV strain “Armenia 2008”. Although ASFV p72-specific IgG antibodies were detectable in all vaccinated animals prior challenge, no protection could be observed. All animals developed an acute lethal course of ASF and had to be euthanized at a moderate humane endpoint within six days. Indeed, the vaccinated pigs showed even higher clinical scores and a higher inner body temperature than the control group. However, significantly lower viral loads were detectable in spleen and liver of immunized animals at the time point of euthanasia. This phenomenon suggests an immune mediated disease enhancement that needs further investigation.

## Introduction

The causative agent of African swine fever (ASF) is a large double-stranded DNA virus (1, 2) which belongs to the *Asfivirus* genus of the *Asfarviridae* family. Over the last decade, the disease has become a pandemic threat to domestic and wild pigs. Overall, more than 55 countries on 5 continents are affected (OIE WAHIS, visited online on October 30^th^ 2021) resulting in tremendous socio-economic losses in the pig industry (3). The virus strains involved in this pandemic belong to p72 genotype II and the vast majority shows high virulence, in both domestic pigs and wild boar. However, strains of lower virulence have been reported from Estonia (4, 5), Latvia (6, 7), and more recently from China (8).

The greatest challenge of ASF control is the development of a safe and effective vaccine (9). Until then, strict biosecurity, early detection, and veterinary hygiene are the only tools to prevent and control the disease.

In the past, many traditional approaches to develop a vaccine against ASFV have failed. Up to now, the most promising vaccine candidates are live attenuated (naturally or by deletion) ASFV. These however, have several disadvantages including long-term side effects in some candidates, safety issues related to genetic stability, the requirement for high biocontainment during production, and the lack of suitable cell lines that can be scaled up without leading to changes in the viral genome. The latter remains a key constraint for production (10).

Inactivated vaccines are most interesting from a safety point of view. Unfortunately, they have not yet been shown to be effective (9). Under the assumption that the integrity of viral particles could enhance and facilitate presentation of antigens that are crucial for protection, conservative inactivation protocols have been discussed in the aftermath of different ASF research projects. Among different options of inactivation, gamma irradiation, in combination with a strong adjuvant, was considered a promising approach (11) and thus it was the chosen method for this study. The advantage of inactivation by gamma irradiation is that it damages only the DNA and RNA while preserving both surface antigens and viral structure (12).

Here, we report on a study which was carried out to evaluate the effectiveness of a structure preserving inactivation of ASFV with gamma irradiation and the potential use as a vaccine in combination with two state of the art adjuvants (Polygen™ Adjuvant, MVP Laboratories and Montanide™ ISA 201 VG, SEPPIC).

## Material and Methods

### Experimental Design

The study included 15 domestic pigs (German Landrace x Large White) of approximately 8-weeks of age, weighing 20-25 kg, and of both sexes, divided in three equally sized groups. All animals were bought from a commercial pig farm and were clinically healthy upon arrival.

All animals were tested negative for ASFV and ASFV specific antibodies prior to enrollment in the study. The animals were kept in the high containment facility of the Friedrich-Loeffler-Institut (FLI), Isle of Riems, Germany. Over the course of the trial, the animals were fed a commercial pig feed with corn and hay-cob supplement and had access to water *ad libitum*. Enrichment material of different matrices was offered over the entire experimental time.

After an acclimatization phase, the domestic pigs were vaccinated intramuscularly with 2 ml of the respective adjuvanted preparation.

Clinical parameters of all animals were assessed daily based on a harmonized scoring system as previously described (13). The sum of the points was recorded as the clinical score (CS) that was also used to define the humane endpoint which was set at 10 clinical score points (moderate humane endpoint).

Blood samples were collected at day (d) 0 prior to vaccination, and at d7, d14, d21, d28, d35, and d42 after the first vaccination. On day 21 after the first vaccination, the pigs received a booster vaccination. On day 42 after the first vaccination/21 days after the second vaccination, the animals were challenged oro-nasally with a highly virulent ASFV genotype II isolate (ASFV “Armenia 08”). After the challenge, blood samples were collected at 4 days post challenge (dpc) and at the day of euthanasia (6 dpc for vaccinated groups and 7 dpc for control group). Animals that reached the humane endpoint or that suffered unacceptably without reaching the endpoint were euthanized through intracardial injection of embutramide (T61, Merck, Darmstadt, Germany) under deep anaesthesia with tiletamine/zolazepam (Zoletil^®^, Virbac, Carros, France), ketamine (Ketamin 10%, Medistar, Ascheberg, Germany) and xylazine (Xylavet^®^ 20mg/ml, CP-Pharma, Burgdorf, Germany) or ketamine (Ketamin 10%, Medistar, Ascheberg, Germany) and azaperone (Stresnil™ 40mg/ml, Elanco, Bad Homburg, Germany).

Necropsy was performed on all animals to evaluate ASFV induced lesions. Lesions were scored based on the protocol published by Galindo-Cardiel et al. (14) with slight modifications (15). Tissue samples (spleen, kidney, liver, tonsil, bone marrow and lymph nodes) were taken for further analysis and reference material acquisition.

Over the entire study period, all applicable animal welfare regulations, including EU Directive 2010/63/EC and institutional guidelines, were taken into consideration. The animal experiment was approved by the competent authority (Landesamt für Landwirtschaft, Lebensmittelsicherheit und Fischerei (LALLF) Mecklenburg-Vorpommern, Rostock, Germany) under reference number 7221.3-1.1-003/20.

### Cells

Blood for the preparation of peripheral blood mononuclear cell (PBMC)-derived macrophages was collected from healthy domestic donor pigs that were kept in the quarantine stable at the FLI. In brief, PBMCs were obtained from EDTA-anticoagulated blood using Pancoll animal density gradient medium (PAN Biotech, Aidenbach, Germany). PBMCs were grown in RPMI-1640 cell culture medium with 4-(2-hydroxyethyl)-1-piperazineethanesulfonic acid (HEPES) and 10% foetal calf serum (FCS) at 37°C in a humidified atmosphere containing 5% CO_2_. The medium was supplied with amphotericin B, streptomycin and penicillin to mitigate bacterial and fungal growth. To facilitate maturation of macrophages, granulocyte macrophage colony-stimulating factor (GM-CSF) (Biomol, Hamburg, Germany) was added to the cell culture medium at a concentration of 2 ng/ml. The cells were used for virus cultivation and hemadsorption test (virus isolation and titration).

### Virus Material

The genotype II ASFV isolate “Estonia 2014” (5) was used for gamma irradiation and subsequent vaccination. A virus stock was prepared in PBMCs and the titer was determined by hemadsorption test as previously described (16). The titer amounted to 10^7.25^ haemadsorbing units 50% (HAU)/ml. Inactivation of the irradiated virus suspension was verified employing hemadsorption tests.

For challenge infection, a spleen suspension containing genotype II ASFV “Armenia 2008” was used with a final titer of 10^6^ HAU per ml. The titer was confirmed by end-point back titration of the inoculum with hemadsorption test.

### Gamma Irradiation

The virus stock of ASFV “Estonia 2014” was mixed with 25% of trehalose before irradiation to protect the virus structure during the process. The irradiation was performed with the Model 812 Co-60 irradiator (Foss Therapy Services, Inc., California, USA) in a frozen condition at -80°C where the vials were placed in a Bio bottle with dry ice. For the calculation of the dose and estimation of the time, the GAMMA FOSS Spreadsheet and Dose Tracker software (California, USA), which are connected to the gamma irradiator machine, were used. The software calculates the time of exposure needed based on the emission rate of the Cobalt60 source.

For determination of the inactivation dose, the virus samples were irradiated at 2, 4, 5, 6, 7, 8, 10, 20, 30, 40 and 50 kGy. For the irradiation of the vaccine, a dose of 30 kGy was chosen based on the internationally accepted standard sterility assurance level (SAL) which is 6 times of the D_10_ value. The D_10_ value is the ability of gamma irradiation to reduce an exposed microbial population by 90 per cent (one log10) under standard conditions of time, temperature and dose. The D_10_ value of ASFV “Estonia 2014” vaccine formulation was calculated using the inverse of the slope of the regression lines (-1/slope) of gamma irradiation dose against log virus titer using GraphPad prism 9 (17).

### Transmission Electron Microscopy

At 30 kGy irradiated cell culture supernatant containing ASFV supplemented with 25% trehalose was prepared for electron microscopy by negative staining technique as described by Rubbenstroth et al. (18) and analysed in a JEM 1011 transmission electron microscope (JEOL, Freising, Germany) at 80 kv and 200,000-fold magnification.

### Adjuvants

Polygen™ (MVP Laboratories, Inc. Omaha, USA) is a low molecular weight copolymer adjuvant that has demonstrated to stimulate significant interferon gamma and interleukin 12 responses when used in a parasite vaccine for cattle (19). Moreover, it has recently been used successfully with an inactivated large DNA virus, i.e. Capripox virus (20).

Montanide™ ISA 201 VG (Seppic, La Garenne-Colombes, France) is a Water-in-Oil-in-Water (W/O/W) formulation, that is a continuous aqueous phase emulsion in which droplets of oil contain a secondary aqueous phase (double emulsion). Due to their structure, they can induce a short and long-term protective immune response. Field trials have shown that such adjuvant can stimulate both humoral and cell mediated immune responses (21).

### Preparation of the Vaccine

#### 30 kGy Irradiated ASFV With Polygen™ Adjuvant

For the preparation of the vaccine, the irradiated virus suspension was combined with 20% of Polygen™ adjuvant. For this, the Polygen™ adjuvant was gently mixed for 2 h before the antigen was added to the suspension. During addition of the antigen, the suspension was gently mixed with a magnetic stirrer and stirring was continued for 2 h after complete addition of the antigen. Syringes were filled with 2 ml of the prepared vaccine and stored on ice until further use.

#### 30 kGy Irradiated ASFV With Montanide™ ISA 201 VG

To prepare the vaccine, both phases, the virus suspension and the Montanide™ ISA 201 VG adjuvant, needed to be combined with a 50/50 w/w ratio. The adjuvant was sterilised by 0.2 µm filtration. Both phases were warmed to 32°C. The virus suspension was then slowly added to the adjuvant under magnetic agitation at 32°C. After the addition of the entire volume, the agitation was continued for 5 min at 32°C. After that, the emulsion was cooled down for 1 h to 20°C. Syringes were filled with 2 ml of the prepared vaccine and stored on ice until further use.

### Processing of Samples

Serum samples were aliquoted after centrifugation at 2.500 *x g* for 20 min at 20°C and together with aliquoted EDTA samples stored at -80°C until further use.

Tissue samples, which were collected during necropsy, were stored at -80°C. For further processing, tissue samples (a lentil-sized piece) were homogenized in 1 ml phosphate-buffered saline (PBS) with a metal bead using a TissueLyser II (Qiagen^®^GmbH, Hilden, Germany) at 30 Hz for 3 min before extraction and qPCRs were performed.

### Virus Detection

For qPCR, viral nucleic acids were extracted from 100 µl tissue homogenate using the NucleoMag Vet Kit (Machery-Nagel, Düren, Germany) and the KingFisher^®^ extraction platform (Thermo Scientific, Schwerte, Germany) according to the manufacturer’s recommendations. qPCRs were performed according to the protocol published by King et al. (22) with slight modifications (addition of a heterologous control DNA). All PCR runs were performed using a C1000™ thermal cycler (BIO-RAD, Hercules, California), with the corresponding CFX96™ Real-Time System. Results of all qPCR runs were recorded as quantification cycle (cq) values. A cut off >42 was defined for negative results. Using a dilution series of an ASFV DNA standard, genome copy (gc) numbers were estimated.

To verify the integrity of the p72 antigen in the irradiated virus suspension, the Ingezim ^®^ ASFV CROM Ag (Eurofins Technologies Ingenasa) lateral flow assay, which is a double antibody sandwich immunochromatographic assay for the detection of ASFV antigen in blood samples (23) was used. The test procedure was conducted according to the manufacturer’s instructions.

### Antibody Detection

Sera were tested for the presence of ASFV p72-specific antibodies with the commercially available competitive INGEZIM PPA COMPAC ELISA (Ingenasa, Spain). Additionally, serum samples were tested with an indirect immunoperoxidase test (IIPT) according to standard protocols provided by the European Union Reference Laboratory for ASF (EURL protocol: https://asf-referencelab.info/asf/images/ficherosasf/PROTOCOLOS-EN/2021_UPDATE/SOP-ASF-IPT-1_2021.pdf (accessed on 7 January 2021)) with slight modifications.

### Data Analysis

All data were recorded and evaluated using Microsoft Excel 2010 (Microsoft Deutschland GmbH, Munich Germany).

GraphPad Prism 9 (Graphpad Software Inc., San Diego, CA, USA) was used for statistical analysis and graphs. Statistically significant differences were investigated by multiple t-tests with Holm-Sidak’s correction for multiple comparisons and with ordinary one-way ANOVA for viral genome detection in blood and organ samples between the groups. Statistical significance was defined as p < 0.05 and indicated with an asterisk (*).

## Results

### Determination of the Inactivation Dose With Irradiation and Integrity of ASFV

In order to determine the exact dose of inactivation, the virus-trehalose suspension was irradiated with different irradiation doses. To quantify the titer reduction and/or to confirm complete inactivation, end-point titrations were performed in quadruplicate (**Figure S1A**) using the hemadsorption test as readout. After an irradiation with 8 kGy, no hemadsorption could be detected. The samples still tested positive by antigen lateral flow device (data not shown) showing an integrity of the p72 antigen.

To account for a substantial safety margin, an irradiation dose of 30 kGy was used in downstream experiments according to the internationally accepted standard sterility assurance level (SAL) which is 6 times of the D_10_ value. The D_10_ was found to be 1.81 kGy (**Figure S1B**).

**Figure 1:**
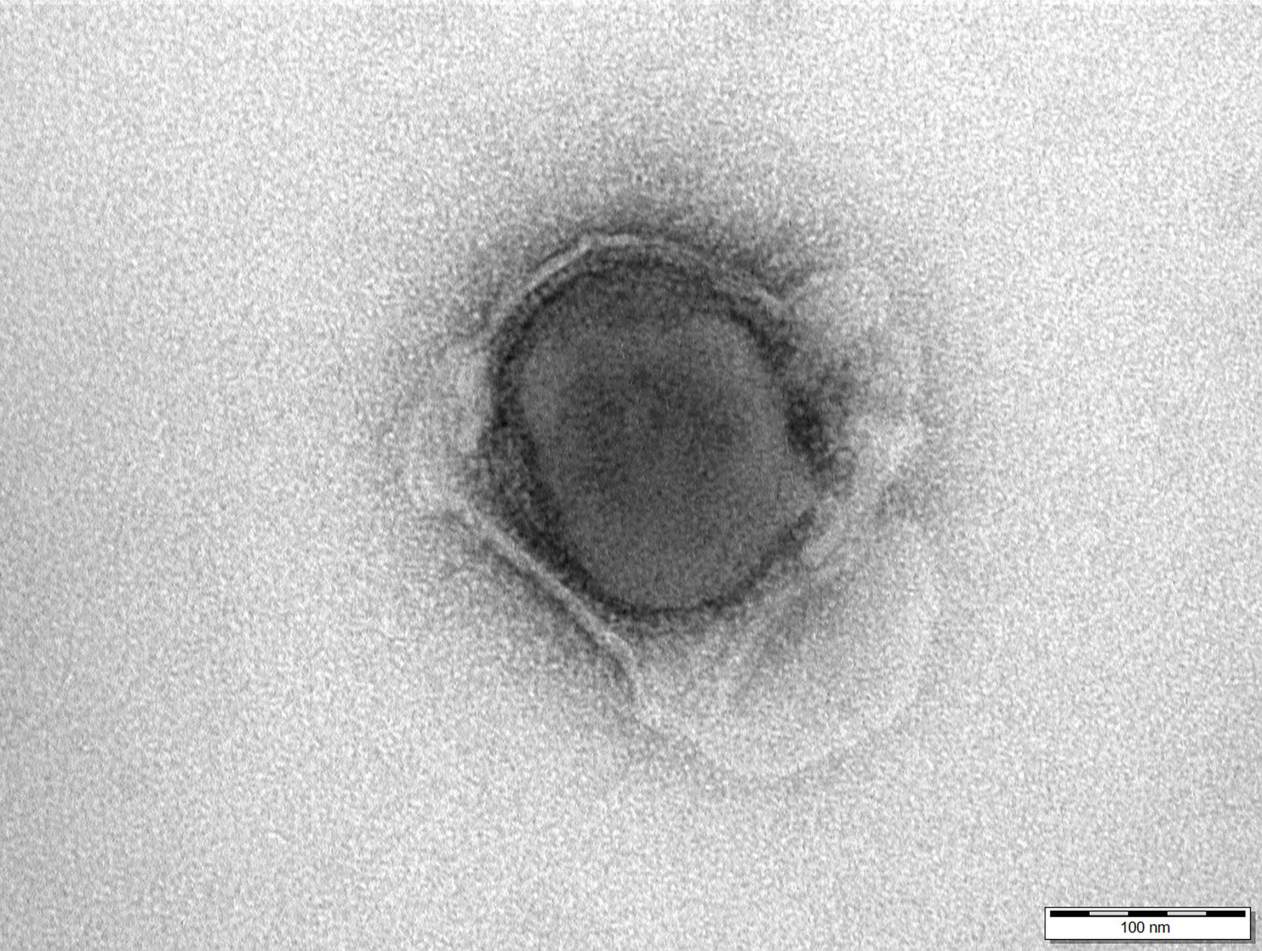
Electron micrograph of a 30 kGy irradiated African swine fever virus shows the integrity of the particle. Negative staining with 1% phosphotungstic acid. Scale bar 100 nm.

The structural integrity of the 30 kGy irradiated and inactivated ASFV particles was confirmed with transmission electron microscope as shown in **Figure 1**.

### Clinical Findings

Vaccination with irradiated ASFV supplemented with Polygen™ or Montanide™ ISA 201 VG was not associated to adverse reactions except for local erythema between one and two cm in diameter in two out of five animals at the intramuscular injection site after the second vaccination which resolved after three to four days. This lesion was more pronounced in the group that received the vaccine with Montanide™ ISA 201 VG.

**Figure 2:**
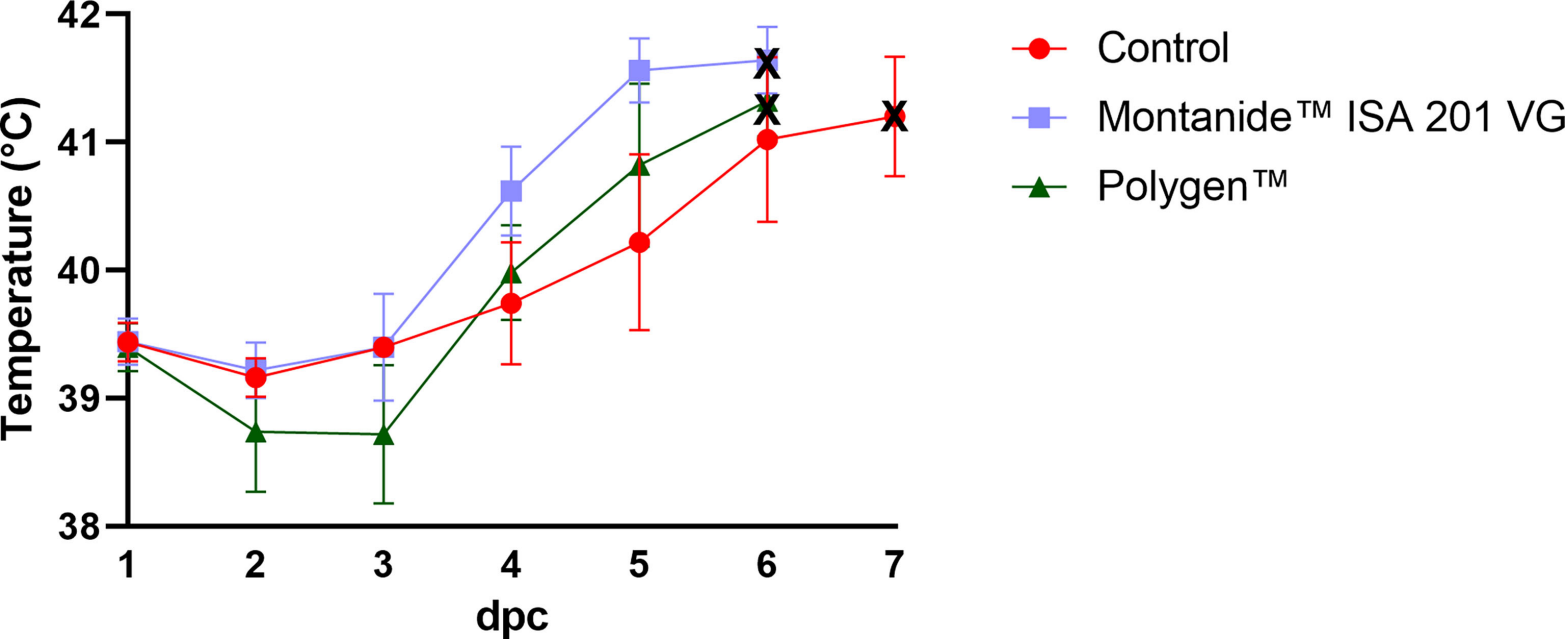
Inner body temperatures depicted as group mean values after challenge (bars indicate standard deviation). Black crosses indicate the day at which the animals reached the moderate humane endpoint. dpc, days post challenge.

**Figure 3:**
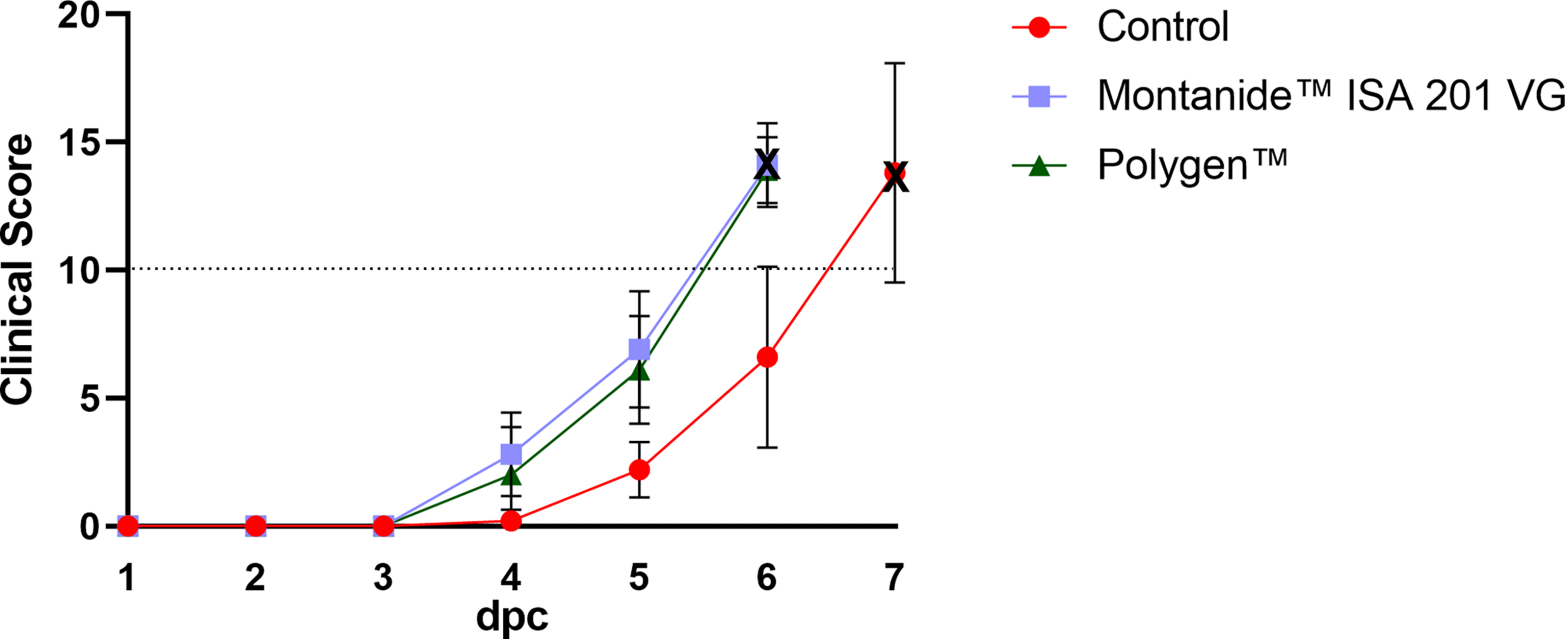
Mean clinical scores (bars indicate standard deviation) of the vaccinated and control groups after challenge. The control group showed a later onset compared to both vaccinated groups. Dotted line indicates the moderate humane endpoint of 10 score points (indicated as dotted horizontal line). The cross marks the day of euthanasia. dpc, days post challenge.

To test whether the vaccines were protective, vaccinated pigs and unvaccinated control animals were challenged oro-nasally with 10^6^ HAU per ml ASFV strain “Armenia 2008”. All animals, whether vaccinated or not, developed severe, unspecific clinical signs starting on day 4 post challenge (dpc). Clinical signs included fever, general depression, lack of appetite, curved back, ataxia, increased lying and respiratory distress. Pigs of all groups developed inner body temperatures higher than 40.0°C from day 4 pc (**Figure 2**). Clinical signs worsened more rapidly in the vaccinated animals compared to the control group. The vaccinated groups reached the humane endpoint of 10 score points at 6 dpc while the control group was sacrificed one day later on 7 dpc. The clinical score for each group is shown in **Figure 3**. There was no marked difference between the vaccinated groups and the control group in the final clinical score points at day of euthanasia (humane endpoint).

### Virus and Viral Genome Detection

**Figure 4:**
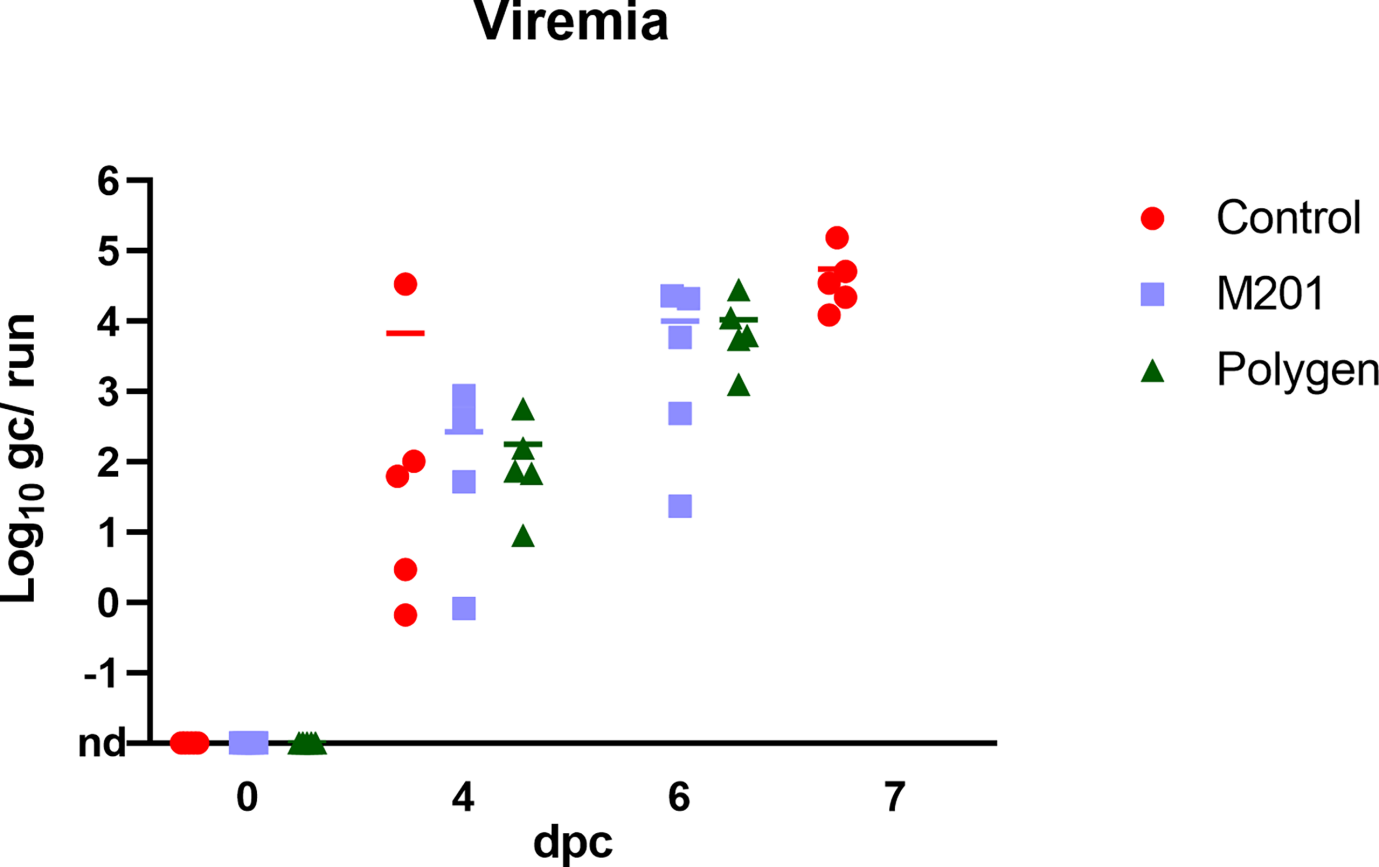
Detection of viral genome in blood samples after challenge infection. Challenge was performed on day 42 after the first vaccination or 21 days after the second vaccination. Blood samples were taken on day 4 pc and at day of euthanasia. Results are expressed as log 10 genome copies (gc)/run. nd, not detected.

**Figure 5:**
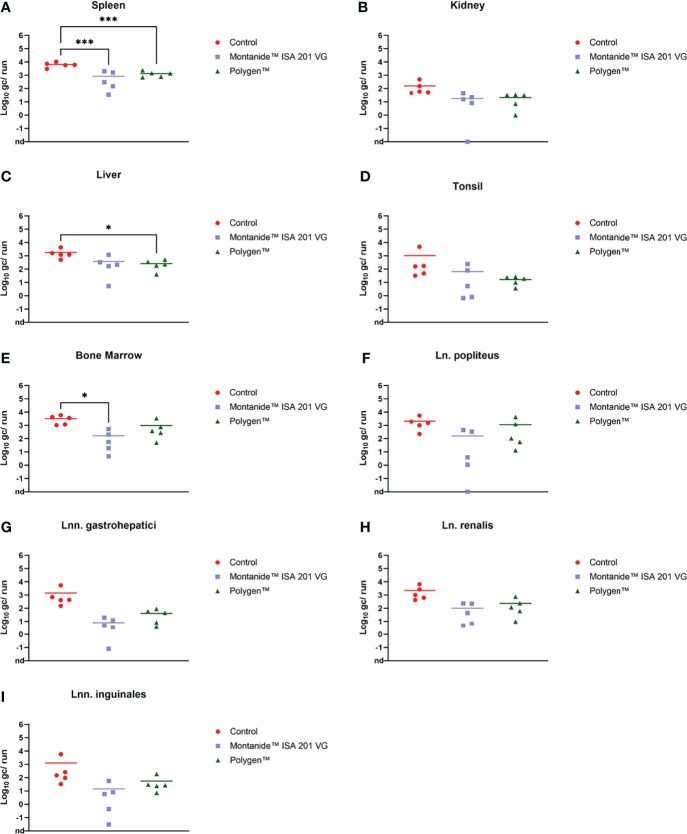
Individual log 10 genome copies (gc)/run obtained by qPCR (King et al., 2003) from spleen **(A)**, kidney **(B)**, liver **(C)**, tonsil **(D)**, bone marrow **(E)**, Ln. popliteus **(F)**, Lnn. gastrohepatici **(G)**, Ln. renalis **(H)** and Lnn. inguinales **(I)** of the vaccinated and control groups. *p-Values: < 0.1; ***p-Values: < 0.001. nd, not detected.

Prior to inoculation, all animals were tested negative for ASFV. After vaccination, no ASFV genome could be detected in blood (data not shown). At 4 dpc, ASFV genome was detectable in blood samples (**Figure 4**) of all groups. In contrast, in one animal (#351) which received Montanide™ ISA 201 VG as adjuvant no viral genome was detectable in blood and only very low genome copies were present in some tissue samples (bone marrow, liver, spleen, lung and Ln. renalis). The vaccinated groups were euthanized on 6 dpc and the control group on 7 dpc. Organ samples obtained from all vaccinated and control animals tested positive in qPCR at day of euthanasia (**Figures 5A**–**I**). In the vaccinated groups there was significant less genome detectible in the spleen compared to the control group (*** p-Values: < 0.001) at the humane endpoint. The vaccinated group with Montanide™ ISA 201 VG as adjuvant shows significantly less genome copy numbers in the bone marrow compared to the control group (* p-Values: < 0.1) and the vaccinated group which received Polygen™ as adjuvant showed significant less genome copy numbers in the liver compared to the control group (* p-Values: < 0.1). No correlation was found between viremia and clinical score (data not shown).

### Pathomorphological Findings

**Figure 6:**
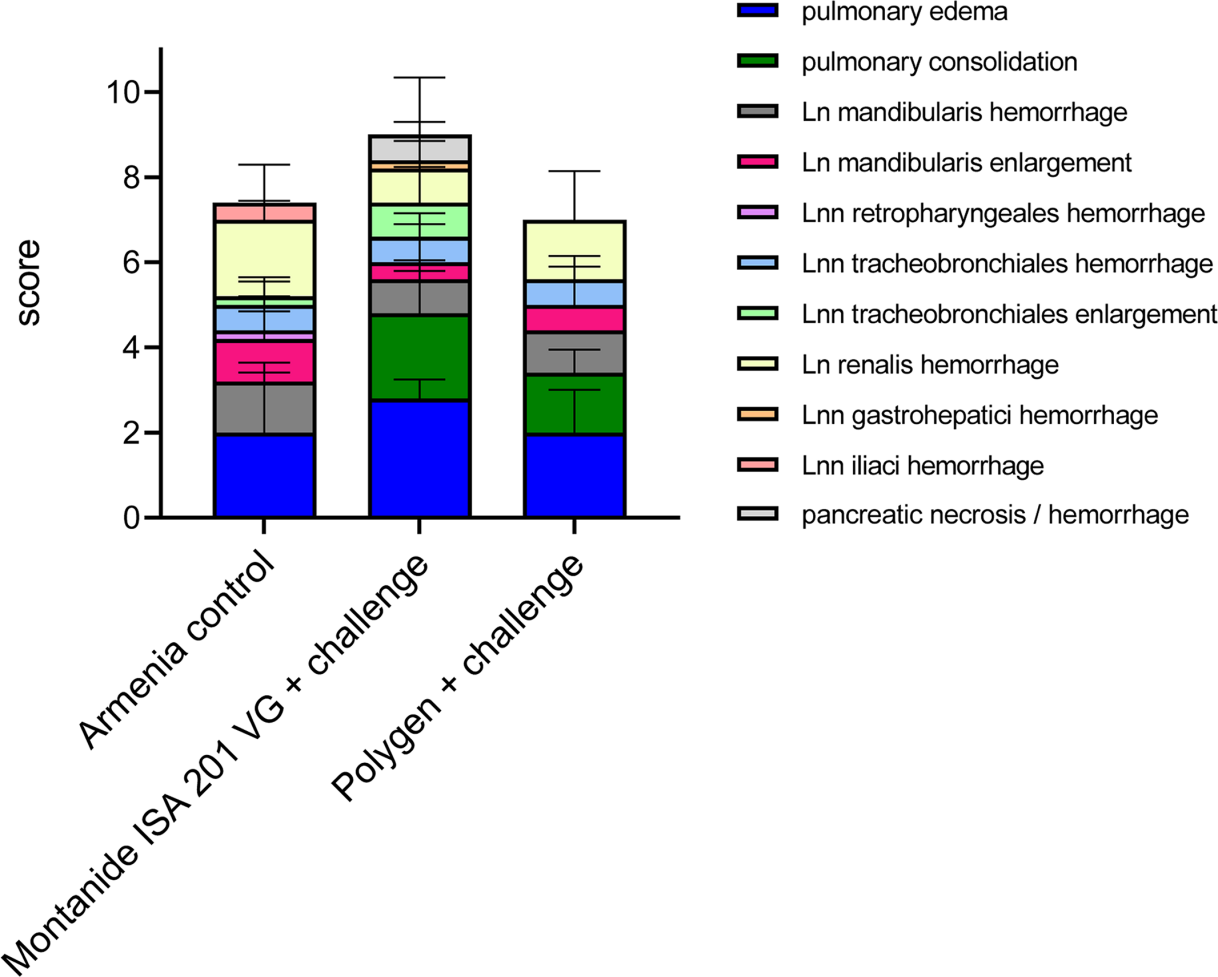
Summary of macroscopic pathological findings in unvaccinated (Armenia control) and vaccinated challenged pigs (Montanide™ ISA 201 VG and Polygen™). The mean values of each individual finding (right legend) from all animals were summarized to form a total score given on the Y-axis.

All animals were subjected to full necropsy. Results of macroscopic scoring are shown in **Figure 6**.

Pigs infected with the highly virulent ASFV strain “Armenia 2008” displayed initial ASF lesions (pulmonary edema and enlarged and hemorrhages lymph nodes) typical for a moderate humane endpoint (sum of 10 score points) decision. Incipient hemorrhages and slight to moderate enlargement were continuously observed for the renal and mandibular lymph nodes, and to a lesser extent for the retropharyngeal, tracheobronchial and iliac lymph nodes. Four out of five pigs suffered from mild to severe pulmonary alveolar edema.

Comparable lesions were present in the animal group vaccinated with irradiated ASFV supplemented with Montanide™ ISA 201 VG followed by a challenge infection with ASFV “Armenia 2008”. Pulmonary edema, but also pulmonary consolidation of varying severity occurred in all animals, while lymph node lesions were less pronounced when compared to control animals. One pig (#312) developed severe pancreatic necrosis and hemorrhage. Although no viremia was detected in animal #351, pathologic findings did not differ from those of other animals in this group.

Likewise, in the animals that received the vaccine supplemented with Polygen™ and challenged with ASFV strain “Armenia 2008”, pathologic changes were indicative for an ASF infection and included mainly mild to severe lymph node hemorrhages, pulmonary edema and consolidation in all affected pigs. No correlation was found between viremia and pathological findings (data not shown).

### Antibody Detection

**Figure 7:**
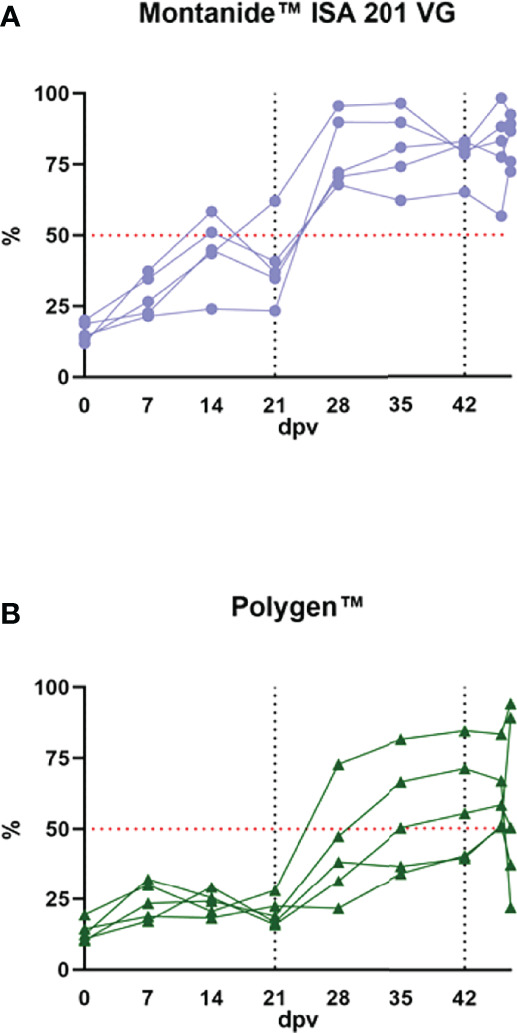
Antibody detection in the vaccinated groups. **(A)** the vaccinated group with Monatide™ ISA 201 VG as adjuvant and **(B)** the vaccinated group with Polygen™ as adjuvant. The ELISA cut off is 50% blocking which is indicated as a red dotted line in both graphs. The first black dotted line indicates the second vaccination and the second dotted line is the time point of the challenge infection.

No antibodies were found in the sera prior to inoculation in any of the samples tested *via* ELISA or in the indirect immunoperoxidase test (IIPT). Fourteen days after the first vaccination, antibodies were detected with ELISA in two animals that received the Montanide™ ISA 201 VG adjuvanted vaccine. After the booster vaccination, all animals of this group seroconverted (**Figure 7A**). In the group with Polygen™ as adjuvant the first animals (3/5) with detectible antibodies in the ELISA were found on day 28 post vaccination. On day 46 post vaccination (4 dpc) all animals showed seroconversion (**Figure 7B**). With the IIPT (data not shown), the first antibody positive animals from the Montanide™ ISA 201 VG group could be detected on 7 days post vaccination (dpv) (3/5, 1 questionable). From 14 dpv all animals were positive. In the Polygen™ group only one animal showed a positive immune response on 7 dpv, and on 14 dpv three animals were positive and two questionable. From 21 dpv all animals from the Polygen™ group were antibody positive. No correlation was found between viremia and antibody detection or between clinical score and antibody detection (data not shown).

## Discussion

The current ASF pandemic endangers animal health and all branches of global pig industry (24). In the absence of vaccines or treatment options, controlling ASF is proving nearly impossible in many regions of the world (25), and research towards vaccines has been intensified.

For the domestic pig sector, safety is a key requirement and thus, inactivated vaccines, vectored vaccines, and subunit approaches would be favorable. Unfortunately, there has been little success in this direction (3).

In an additional attempt of testing an inactivated vaccine, we explored the use of gamma-irradiation for virus inactivation. After the development of gamma irradiators that can provide precise doses, this technique has been used to develop a variety of proof-of-concept vaccine types. The main advantage of this inactivation technique is its ability to destroy nucleic acids of the pathogen while preserving the proteins and thus the antigenicity. Chemical inactivation which is more frequently used in the current inactivated vaccine production, leads to an increased destruction of pathogen proteins compared to radiation-inactivation. Moreover, following radiation-inactivation, there is no need to remove any chemical residues which increases the safety of the vaccine and makes the whole process simpler and less time-consuming (26). Thus, in this study we aimed at preserving the structure of the virus and the natural presentation of crucial antigens. In addition, we chose two adjuvants with different modes of action, namely “Montanide™ ISA 201” and “Polygen™”, that are both known to elicit not only humoral but also cellular responses (20, 21, 27–30). The latter are known to be crucial for protection (31).

In the presented study, inactivation was achieved with doses from 8 kGy onwards with an initial virus titer from 10^7.25^ HAU/ml. Given the impact of the disease and biosafety requirements, a considerable safety margin according to the internationally accepted standard SAL was added, and vaccination was carried out with suspensions irradiated with 30 kGy. This dose is still lower than the dose of 50 kGy that was used by McVicar et al. (32) for bulk samples (titers of the selected tissues ranged from 10^4.6^ to 10^7.8^ HAd_50/_g). However, McVicar (32) showed that an irradiation dose of 20 kGy was sufficient to inactivate ASFV. The latter is in line with more recent studies by Boudarkov et al. (33), who showed that irradiation doses of 20 kGy and higher completely inactivated ASFV with an initial titer of 10^6^ HAd_50_/cm^3^. In the experiment presented here, the infectivity of the irradiated virus was tested by haemadsorption test and complete inactivation was confirmed in the animal trial (no detection of virus in vaccinees). In addition, integrity of the virus particles was confirmed by electron microscopy.

General immunogenicity was shown as all animals developed antibodies against ASFV p72. In detail, the group that received Montanide™ ISA 201 VG as adjuvant, seroconverted earlier and showed higher ASFV p72 antibody levels when compared to the group that received the vaccine with Polygen™ as adjuvant. Unfortunately, challenge infection showed again that no protective immunity was induced. All vaccinated animals reached the humane endpoint even earlier than the controls and displayed typical ASF lesions. However, lower viral loads were detected in organ samples of the vaccinated animals, especially in spleen, liver and bone marrow. This could be due to a partial protection of antibodies, as the level of antibodies decreased slightly after challenge, indicating consumption. Another explanation could be the fact that the vaccinees had to be euthanized one day earlier than the control group. Jancovich et al. (34), also observed that if animals were vaccinated, decreased levels of ASFV genome in blood and some soft tissues were found after challenge compared to those in control pigs. One pig (# 351) from the Montanide™ ISA 201 VG adjuvanted group showed even no viremia at 4 dpi and very low genome copies in organ samples at the day of euthanasia, although the clinical signs were severe. It cannot be completely ruled out, however, that technical problems during challenge or sampling could have led to the observed difference for this particular pig. These findings of an earlier onset of clinical signs, viremia, and death in vaccinated animals were in line with other studies that showed lacking protective effects in the presence of high antibody levels (9, 35, 36).

The above indicated slightly accelerated clinical course in vaccinated animals in the absence of higher virus replication could indicate an immune-mediated disease enhancement of so far unknown genesis. This phenomenon was also seen in the study with inactivated preparations by Blome et al. (35) and in the study by Lokhandwala et al. (37), which used an Adeno-vectored vaccine. Experiments with DNA vaccines and recombinant proteins have also led to an earlier onset of clinical signs, viremia and death in vaccinated animals after challenge (34, 38, 39). Enhanced susceptibility to certain virus infection due to pre-existing immunity can occur through mechanisms involving antibodies, activated macrophages, CD4+T-cells, and dendritic cells (40, 41). One such mechanism, antibody-dependent enhancement (ADE), is a well-documented phenomenon for viral pathogens such as dengue virus and HIV (42, 43), but also MERS-CoV or SARS-CoV spike S protein (44). For ASFV, the underlying mechanisms remain to be elucidated.

Since this was a proof-of-concept study, groups of animals were restricted for this study in terms of the adjuvants, direct comparison was beyond the scope of our study and we did also not include an adjuvant-only or vaccine-only control. In the end this hampers full evaluation of enhancing effects, but as all animals succumbed, follow-up studies may not be indicated. Taking published studies into account, a detrimental effect of powerful adjuvants cannot be completely excluded.

The route of vaccine administration can also play an important role and is worthy of further research in the context of immunization protocols. For example, it was observed that pigs infected with the naturally tick attenuated genotype I OURT88/3 virus were protected against virulent wild type OURT88/1 challenge when administered at low to intermediate doses 10^3^–10^4^ pfu intranasally, but not when administered intramuscularly at the same doses (10). Another route that could be beneficial is the intra-dermal application as antigen-presenting dendritic cells are highly abundant in dermal tissues (45).

Based on the negative results of the use of inactivated vaccines against ASF from previous studies and this one, the use of inactivated vaccines against ASF does not seem to be a viable strategy to date. The lack of neutralizing antibodies plays a major role in the development of an effective inactivated vaccine (9). The insufficiency of inactivated vaccines, along with the lack of efficacy of subunit vaccines, can be explained by the fact that cellular immunity plays a crucial role in protection against ASFV (46, 47). To generate a cellular response, there should be viral replication in the host, which explains effectiveness of live attenuated vaccines (9).

In summary, it can be said that ASF virus inactivated by gamma irradiation cannot be used as a vaccine due to the lack of protection after challenge. The phenomenon of significantly lower viral loads in spleen and liver of immunized animals at the time point of euthanasia, which suggests an immune mediated disease enhancement, needs further investigation.

## Data Availability Statement

The raw data that support the findings of this study are available from the corresponding author upon reasonable request.

## Ethics Statement

The animal study was reviewed and approved by Landesamt für Landwirtschaft, Lebensmittelsicherheit und Fischerei (LALLF) Mecklenburg-Vorpommern, Rostock, Germany; reference number 7221.3-1.1-003/20.

## Author Contributions

Conceptualization, JP, VW, GC, KP, MB, and SB. Methodology, VA, KP, HR, JP, JS-E, LP, and RK. Formal analysis, VA, KP, JP, HR, LP, VW, and SB. Investigation, KP, JP, HR, JS-E, LP, and RK. Resources, VW, GC, MB, and SB. Data curation, VA, KP, JP, HR, LP, and JS-E. Writing—original draft preparation, JP, LP, and SB. Writing—review and editing, GC, SB, and MB. Visualization, JP, HR, LP, and SB. Supervision, VW, GC, SB, and MB. Project administration, JP, VW, MB, and SB. Funding acquisition, GC and SB. All authors have read and agreed to the published version of the manuscript.

## Conflict of Interest

The authors declare that the research was conducted in the absence of any commercial or financial relationships that could be construed as a potential conflict of interest.

## Publisher’s Note

All claims expressed in this article are solely those of the authors and do not necessarily represent those of their affiliated organizations, or those of the publisher, the editors and the reviewers. Any product that may be evaluated in this article, or claim that may be made by its manufacturer, is not guaranteed or endorsed by the publisher.

## Supplementary Material

Supplementary Figure S1 **(A)** Effect of gamma irradiation on the infectivity of ASFV “Estonia 2014”. The mean virus titer is calculated in HAU/ml. **(B)** Plot used for calculating the gamma radiation dose required to reduce infectivity of ASFV by 90% (D_10_ value) in kGy.
