# Supplementary figures and images for: Vaccination With a Gamma Irradiation-Inactivated African Swine Fever Virus Is Safe But Does Not Protect Against a Challenge
Source: Front Immunol. 2022 Apr 26;13:832264. doi: 10.3389/fimmu.2022.832264 (PMC9088005; doi:10.3389/fimmu.2022.832264)

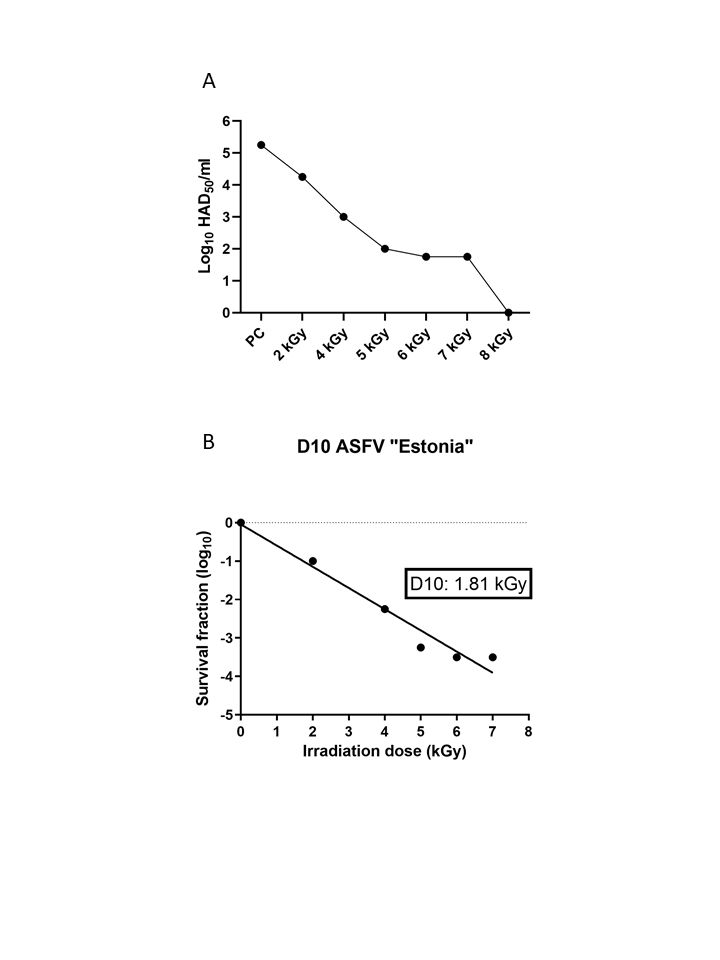

Supplement: Supplementary Figure S1 — (A) Effect of gamma irradiation on the infectivity of ASFV “Estonia 2014”. The mean virus titer is calculated in HAU/ml. (B) Plot used for calculating the gamma radiation dose required to reduce infectivity of ASFV by 90% (D10 value) in kGy. [file Image_1.tif]
